# Response to Bode and colleagues: ‘Resilient reefs may exist, but can larval dispersal models find them?’

**DOI:** 10.1371/journal.pbio.2007047

**Published:** 2018-08-22

**Authors:** Peter J. Mumby, Karlo Hock, Scott A. Condie, Juan C. Ortiz, Nicholas H. Wolff, Kenneth R. N. Anthony, Paul G. Blackwell

**Affiliations:** 1 Marine Spatial Ecology Lab, School of Biological Sciences, The University of Queensland, Brisbane St Lucia, Australia; 2 CSIRO Oceans & Atmosphere, Hobart, Australia; 3 Australian Institute of Marine Science, Townsville, Australia; 4 The Nature Conservancy, Brunswick, Maine, United States of America; 5 School of Mathematics & Statistics, University of Sheffield, Sheffield, United Kingdom

Bode and colleagues’ response to our paper posits that the identification of specific reefs that drive recovery is unsafe because models of larval dispersal (‘connectivity’) are insufficiently accurate or consistent to make useful predictions at this scale [1]. They go on to argue that managers should not base decisions on reef-level predictions of connectivity. The evidence they provide for these assertions is that the results of their own modelling—with a different model but run with a ‘comparable parameterisation’—diverge considerably from ours.

The legitimacy of Bode and colleagues’ concern rests on the apparent disparity in behaviour between connectivity models. Unfortunately, Bode and colleagues’ study differs from ours in many more ways than simply the modelling of oceanography. In short, (1) their representation of the Great Barrier Reef (GBR) excludes approximately half the reefs we recognise (2,175 versus 3,806), so the network metrics of those reefs ‘in common’ are incomparable; (2) they represent different species by ignoring larvae with short settlement times (<7–14 days) and allowing a continuous release of larvae, whereas we considered a wider range of competencies beginning at 12 hours and simulated punctuated spawning events that are more relevant to invertebrates; (3) their model was parameterised two decades ago (1996–2002), so their assessment makes the unlikely assumption that conditions had not changed by the recent period we represented (2008–2013); (4) their model is unable to capture an important dispersal process on the GBR because it takes a two-dimensional depth-integrated approach that ignores the shear flow captured in our 3D Connie model; and (5) they were unable to implement all of the connectivity criteria we applied. Specifically, their finding of a strongly connected network precluded use of graph theory to identify regionally connected sources as we did. The causes of such strong connections in Bode and colleagues’ analysis are unclear but likely include use of higher rates of larval release and a continuous release period of 156 days, though we consider this to be an inappropriate representation of coral spawning, and we elected to retain the temporal dynamics of multiple punctuated spawning events. Given these five fundamental differences, it is not surprising that their assessment of connectivity patterns differed from ours. However, this discrepancy does not imply that choice of hydrodynamic model alone is the cause. Nor does it constitute evidence that our identification of key source reefs is too uncertain to be useful.

The eReefs/Connie hydrodynamic model we adopted has been calibrated against data from tide gauges, wave-rider buoys, an extensive array of moorings and temperature loggers across the GBR, and Argo floats [2] as well as formal assimilation of satellite data. An additional, and probably even more stringent, constraint on the eReefs hydrodynamics was a requirement to drive biogeochemical (BGC) exchanges and generate realistic BGC fields. This entailed calibration and/or validation against an additional 15 long-term data sets (https://research.csiro.au/ereefs/models/). While this model is highly sophisticated, the processes it simulates are naturally variable in time and space. With this in mind, we took steps to identify only the most robust connections among reefs that consistently function as both local and regional sources to many reefs over multiple life-history strategies and seasons. This resulted in identifying 545 source reefs (14% of the GBR) and not <1% as misrepresented by Bode and colleagues.

Oceanographic models differ in their resolution and complexity. We agree that an ensemble approach to modelling is potentially advantageous, but it depends wholly on the appropriateness of the models and on models having complementary skills. For example, we are currently developing a mixed model approach that pairs Connie, which has excellent boundary conditions and 3D stratification of hydrodynamic layers, with the Second-generation Louvain-la-Neuve Ice-Ocean Model (SLIM) [3], which, like Bode and colleagues’ model, is better able to resolve fine-scale larval retention than Connie but does so for the same time period and ecosystem representation as Connie.

The outputs of connectivity modelling can indeed be useful for ecosystem management [4], particularly when models have been tested extensively and steps taken to utilise only the more robust predictions. Yet scientists must convey model limitations and the appropriate context while also continuing to test assumptions and predictions. The use of connectivity models for management usually assumes—as we do—that larval supply is a demographically relevant process. Yet this is not always the case, particularly when post-settlement mortality is high, such as in degraded environments or areas where high adult density constrains juvenile survival [5]. Our study concerned the dual roles of connectivity that consider the recolonisation of corals after the mass mortality from so-called ‘coral bleaching’ and the spread of crown-of-thorns starfish (CoTS) epidemics [6]. In both cases, larval supply is likely to be an essential step of colonisation and recovery, and indeed, our field testing of the CoTS connectivity models was consistent with predictions [6]. Our study highlights the great heterogeneity in the ecological functioning of reefs, with some having far greater potential than others to stimulate regional recovery. Harnessing such heterogeneity to target management interventions should lead to better decisions, which will themselves improve as the usage of connectivity models continues to be refined and improved.

## Abbreviations

BGC: biogeochemical
CoTS: crown-of-thorns starfish
GBR: Great Barrier Reef
SLIM: Second-generation Louvain-la-Neuve Ice-Ocean Model

## References

[1] Bode M, Bode L, Choukroun S, James MK, Mason LB. Resilient reefs may exist, but can larval dispersal models find them? PLoS Biol. 2018; 16(8):e2005964 10.1371/journal.pbio.2005964 PMC6104909 30133437

[2] Schiller A, Herzfeld M, Brinkman R, Rizwi F, Andrewartha J. Cross-shelf exchanges between the Coral Sea and the Great Barrier Reef lagoon determined from a regional-scale numerical model. Cont Shelf Res. 2015;109:150–63.

[3] Thomas CJ, Lambrechts J, Wolanski E, Traag VA, Blondel VD, Deleersnijder E, et al Numerical modelling and graph theory tools to study ecological connectivity in the Great Barrier Reef. Ecol Model. 2014;272:160–74.

[4] Darling E, Cote IM. Seeking resilience in marine ecosystems. Science. 2018;359:986–7. 10.1126/science.aas9852 29496864

[5] Steneck RS, Paris CB, Arnold SN, Ablan-Lagman MC, Alcala AC, Butler MJ, et al Thinking and managing outside the box: coalescing connectivity networks to build region-wide resilience in coral reef ecosystems. Coral Reefs. 2009;28(2):367–78.

[6] Hock K, Wolff NH, Ortiz JC, Condie SA, Anthony KRN, Blackwell PG, et al Connectivity and systemic resilience of the Great Barrier Reef. PLoS Biol. 2017;15(11):e2003355 10.1371/journal.pbio.2003355 29182630 PMC5705071

